# Case Report: Endoscope-Assisted Bilateral Nasal Leech Retrieval in a Dog

**DOI:** 10.3389/fvets.2022.881581

**Published:** 2022-07-29

**Authors:** Manpreet Singh, Amit Kumar, Adarsh Kumar, Som P. Tyagi, Rishu Wadhawan

**Affiliations:** ^1^Masters of Veterinary Surgery and Radiology, CSK Himachal Pradesh Agricultural University, Palampur, India; ^2^Chaudhary Sarwan Kumar Himachal Pradesh Krishi Vishvavidyalaya, Palampur, India

**Keywords:** case report, endoscope, nasal leech, retrieval, epistaxis

## Abstract

A non-descript male dog was presented with symptoms, such as chronic recurrent epistaxis, sneezing, and episodes of nasal leech infestation from the past 3 months. The dog was subjected to basic clinical examination, hemato-biochemistry test, and radiographic examination before rhinoscopy. Rhinoscopy was performed with Karl Storz's 5-mm video-otoscope under general anesthesia. Leech infestation in the nasal cavity was found to be the cause of the epistaxis under rhinoscopic examination. The endoscopic guided retrieval of the leech from both the nostrils was successfully done with help of grasping forceps without any major complication. The video-otoscope-guided retrieval of nasal leeches was quite convenient and also provide the exact localization of the nasal leech.

## Introduction

Leeches are blood-sucking hermaphroditic parasites with variable color and length. These are segmented worms with the presence of two suckers. The leech belongs to the class *Hirudinea* and its infestation is known as *Hirudiniasis*. It acts as a living foreign body or solid parasite in the nasal cavity of dogs which leads to severe nasal congestion. The incidence of nasal leech infestation depends upon the geographical area. The life cycle is initialized with the formation of eggs enclosed in a cocoon, then, these cocoons bind with the underwater surface of water bodies. Finally, the young leeches are emerged out from cocoons. Leech infestation in dogs is mostly during drinking of water and swimming in the rural water stream. The most common symptoms were recurrent epistaxis, sneezing, and wriggling of leech outside the nostril (1). Conventionally, the leeches were removed using saturated salt solution irrigation without direct visualization of the nasal cavity. This method had a variable success rate. Thus, the endoscope-guided retrieval technique was attempted in this particular case for the removal of the leech from both nasal cavities.

## Patient Information

A 6-year-old non-descript male dog weighing 30 kg (66 pounds) was presented with a history of upper respiratory tract problems (nasal problem). The patient belonged to Himachal Pradesh (a hilly area of India).

## Clinical Findings

A six-year-old non-descript dolichocephalic male dog was presented with signs of sneezing, intermittent epistaxis, reverse sneezing, and previous episodes of nasal leech infestation from the past 3 months. The dog was subjected to a rhinoscopy procedure after the basic clinical and hemato-biochemical examination.

## Timeline

Previously nasal leech retrieval was done with the traditional method using saturated saline solution. The described traditional method was done in this dog during his previous appointments with the same complaint of the nasal leech. In this case, the traditional method failed to retrieve nasal leeches even after multiple attempts and decided to perform endoscope-guided retrieval to fix a problem.

## Diagnostic Assessment

**Figure 1:**
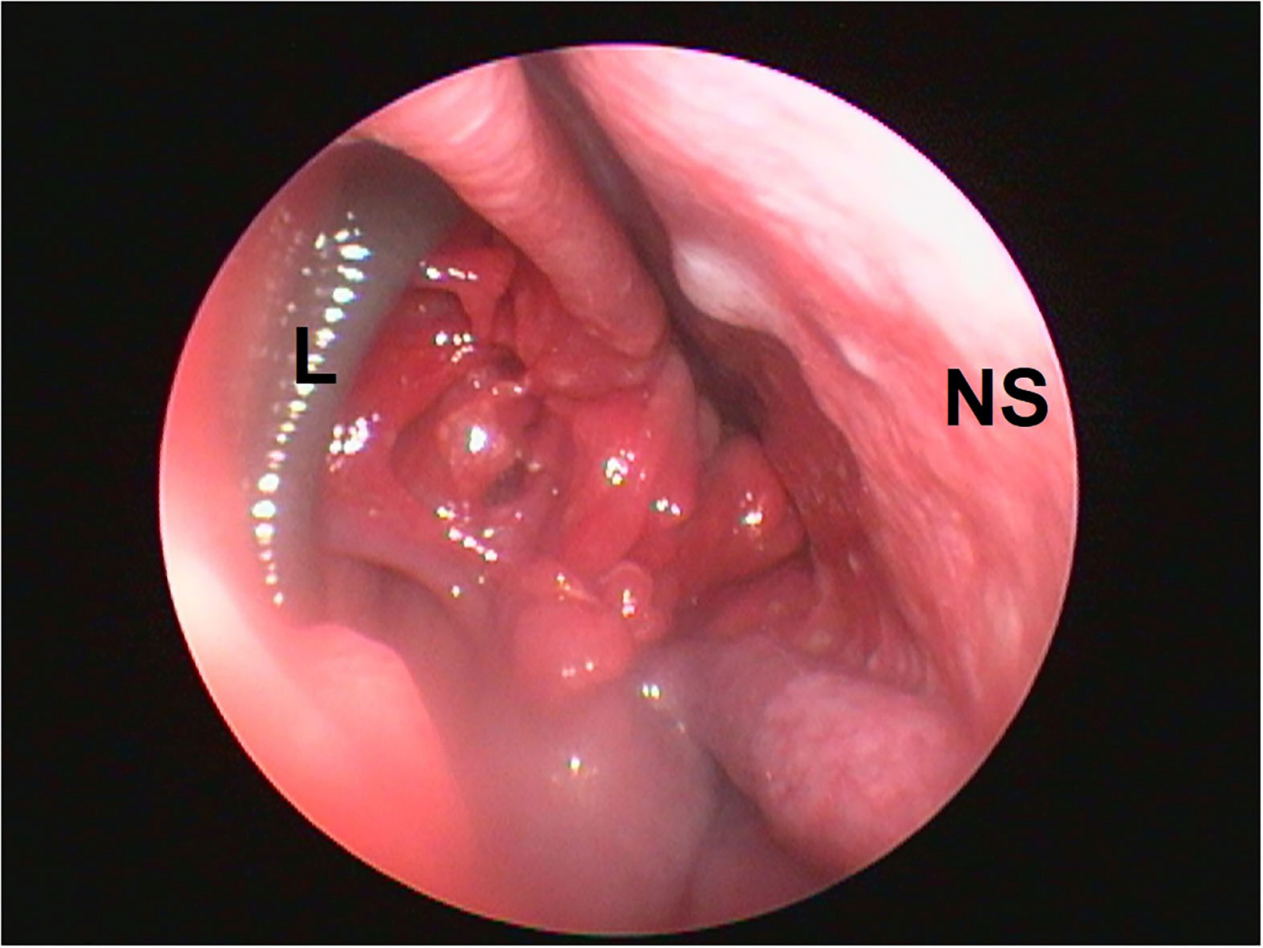
Localization of nasal leech infestation. L, leech; EC, ethmoid conchae; NS, nasal septum.

The physical examination was done to check for nasal bridge elevation to rule out other conditions, such as a nasal tumor, aspergillosis, etc. Physical examination indicates the active status with a rectal temperature of 101.5-degree Fahrenheit and a pale pink conjunctival membrane. The routine lab work, including hemato-biochemistry, revealed reduced hemoglobin and packed cell volume. The rhinoscopy showed the presence of leeches in both the nasal cavities at the level of ethmoid conchae during the second pass of the examination. The nasal mucosa was severely hyperemic along with the presence of several bleeding spots. The nasal mucosa was irregular and friable, along with an altered contour of ethmoid turbinate (Figure 1). In both the nasal canals the leeches adhered to the nasal mucosa at the level of ethmoid conchae. The body of the leeches was dark brown and horizontal striations on their body were observed in this case (Figure 1). It is essential to differentiate a nasal leech infestation from various chronic nasal affections in dogs such as various types of nasal inflammations resulting in nasal bleeding, nasal tumors, nasal mycosis, etc. Nasal tumors and foreign bodies can result in intermittent bleeding, sneezing, and snoring. The nasal bridge elevation in some cases and no history of wriggling of nasal leech outside the external nares can arise the suspicion of nasal tumors.

## Therapeutic Intervention

The nasal leeches were removed endoscopically under general anesthesia. The restraining of the patient for rhinoscopy was done under general anesthesia. The dog was premedicated with Injection Atropine @ 0.02–0.04 mg/kg body weight (I/M or S/C), Injection Butorphanol @ 0.2–0.4 mg/kg body wt. (I/M) and Injection Diazepam @ 0.5 mg/kg body wt. (I/M). Following premedication, induction of anesthesia was done with an intravenous injection of Propofol to the effect, then, the maintenance of general anesthesia was done with a combination of Isoflurane and Oxygen. The procedure was performed in dorso-ventral (sternal) recumbency with the placement of a positioning aid under the neck of the dog because of a bilateral nasal cavity complaint.

The anterior rhinoscopy technique was opted and an autoclavable rigid video-otoscope (Karl Storz 67260 OSA Veterinary otoscope), having a diameter of 5 mm and length of 8.5 cm, was used. The diameter of the working channel of the otoscope was 5 Fr which was utilized for the passage of grasping forceps. The flexible grasping forceps of 34 cm in length and diameter of 5 mm were utilized for grasping the leech. The other supporting instruments which were utilized for image processing and visualization were the tele pack Vet X monitor, single-chip video camera, and fiber-optic light cable.

The rhinoscopy procedure included the visualization of all the three nasal passes as per the standard technique given by Tams and Rawlings (2). The first pass was performed for examination of the dorsal meatus, the second pass for ventral and ethmoid conchae, and the third pass for visualization of the nasopharynx.

**Figure 2:**
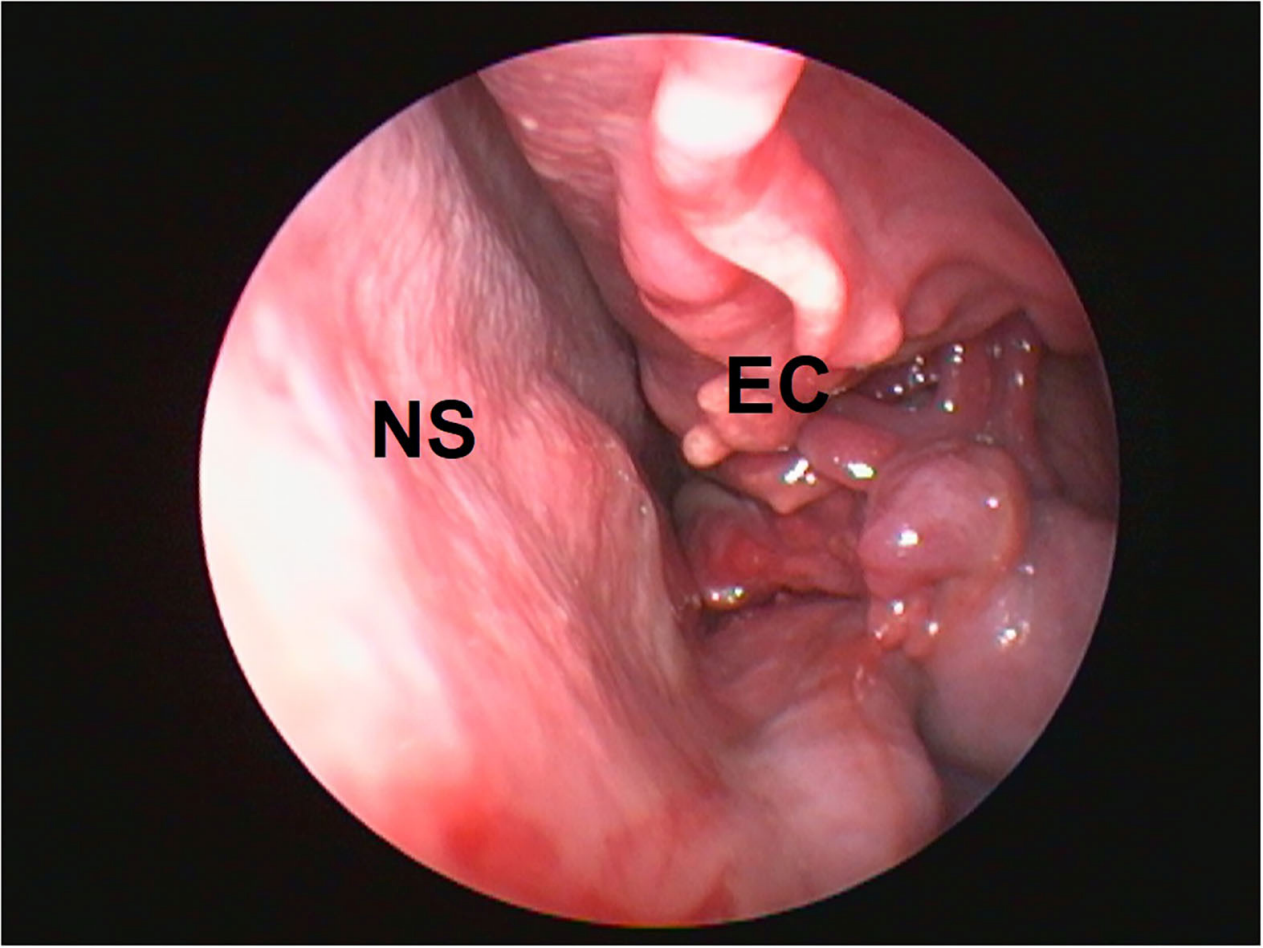
Abnormal contour of ethmoid conchae. EC, ethmoid conchae; NS, nasal septum.

The procedure was done under the surgical plane of general anesthesia because the light plane of general anesthesia was not sufficient for performing rhinoscopy in dogs (3). The rhinoscopic examination revealed the presence of leeches in both the nasal cavities at the level of ethmoid conchae during the second pass of the technique (Figure 1). It was observed that the nasal mucosa was severely hyperemic along with the presence of a few bleeding spots. Moreover, the nasal mucosa was irregular and friable along with an altered contour of ethmoid turbinate (Figure 2). In both the nasal canals the leeches adhered to the nasal mucosa at the level of ethmoid conchae. The body of the leeches was dark brown and horizontal striations on their body were observed (Figure 1).

**Figure 3:**
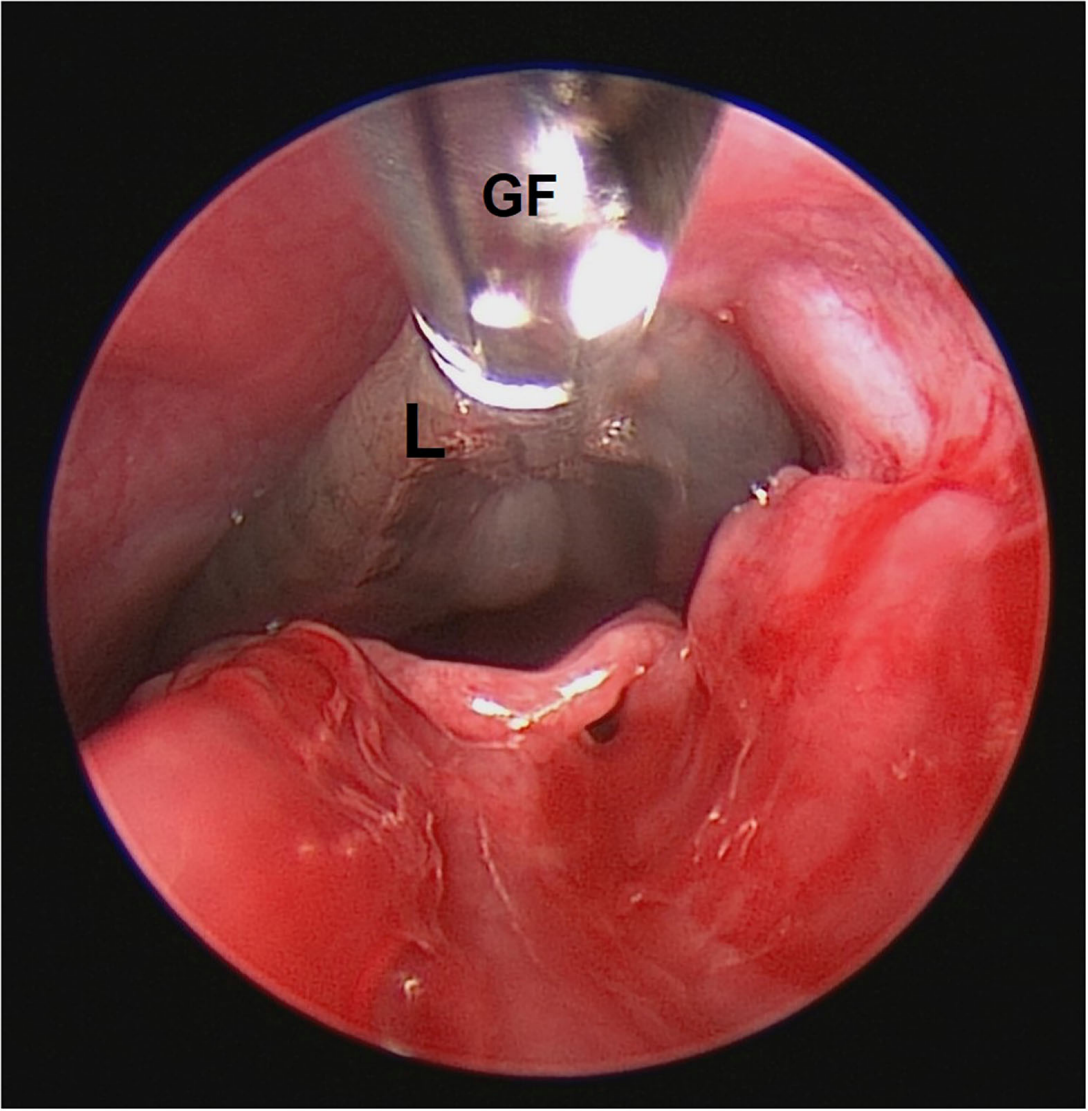
Grasping of leech in nasal cavity. GF, grasping forceps; L, leech.

**Figure 4:**
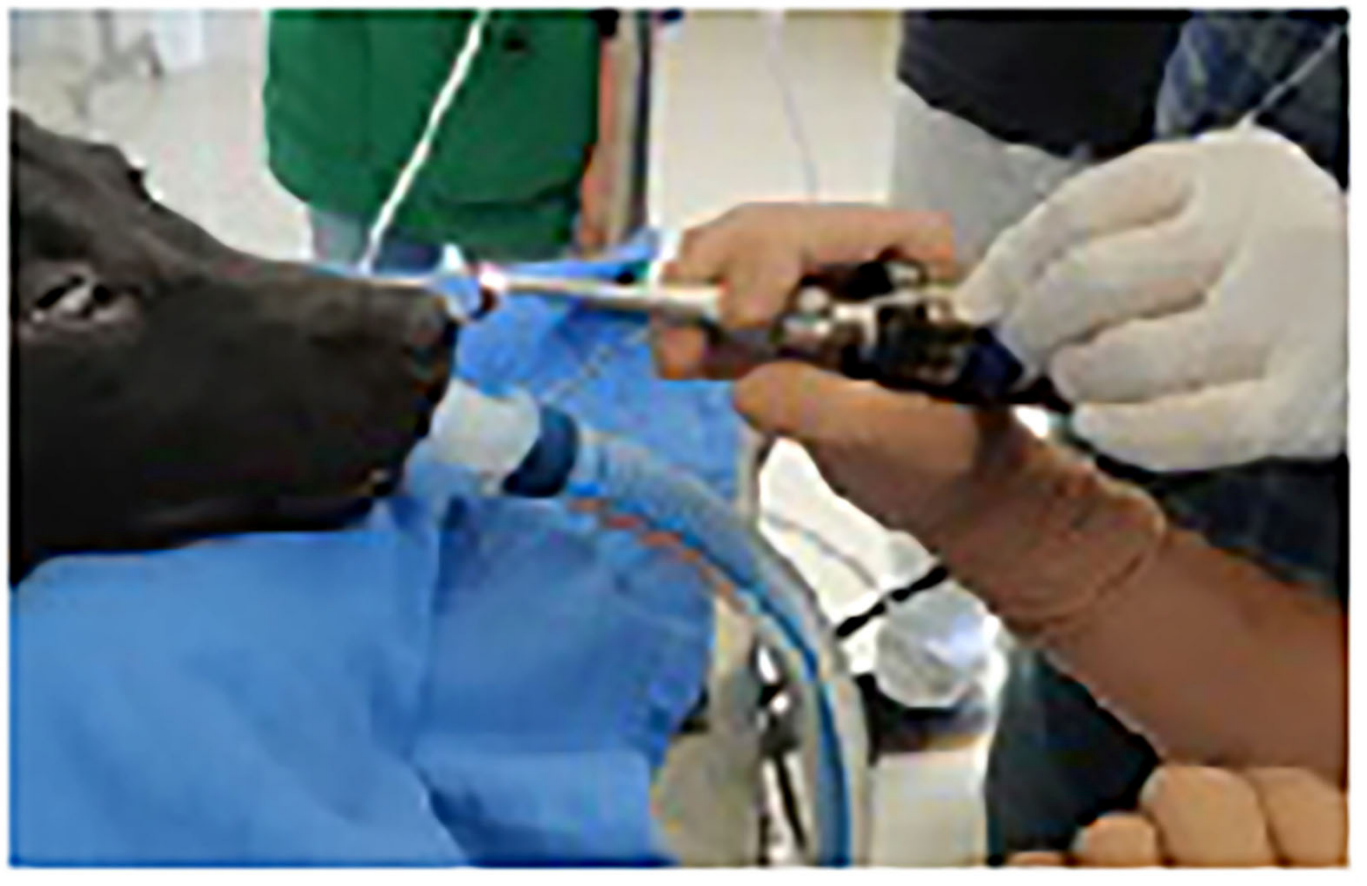
Retrieved nasal leech. RL, retrieved leech; VO, video-otoscope.

Peristaltic movements of leeches within the nasal cavity were also observed. In an attempt to retrieve the leeches, the camera was centered and focused on the body of the leech, and grasping forceps were inserted through the accessory channel of the scope. The body of the leeches was grasped gently (Figure 3), resulting in vigorous movements. The scope and grasped leech were slowly pulled out from the nasal cavity (Figure 3). Finally, the alive and intact leeches were retrieved from both the nasal cavities with help of endoscope-guided grasping forceps (Figure 4). The size of one leech was 10–12 cm and another was 4–5 cm. The smaller-sized leech was not initially seen, but after waiting for a while (Figure 1), peristaltic movements were seen. A dog recovered uneventfully after the whole procedure. The antibiotic (Tablet Clindamycin @ 11 mg/kg body weight twice a day), anti-allergic (Tablet Hydroxyzine @ 2 mg/kg body weight twice a day), and anti-inflammatory drugs (Tablet Carprofen @ 3 mg/kg body weight once a day) were given for 3 days post endoscopically.

## Outcome and Follow-Up

Endoscopic-assisted removal of nasal leeches proved to be beneficial as it gives the exact location of the leech and confirmatory retrieval of the same. The duration of this endoscopic procedure was 5.5 min in this case, which was quite less than the traditional methods. There was no need to put any solutions into the nasal cavities, as well as no apparent complication faced with this technique.

Later on, the patient was presented for reappraisal after 1 week. There was no further nasal bleeding, sneezing, or snoring during the post-endoscopic period.

## Discussion

Leeches are foreign body parasites that can affect the nasal cavity of dogs and cause severe nasal congestion. The most common symptoms were recurrent epistaxis, sneezing, and wriggling of leech outside the nostril (1). The most common technique for nasal leech removal has been the use of the concentrated salt solution, this method has a variable success rate and complications, such as aspiration, irritation to the nasal mucosa, etc. Ralte (4) removed the nasal leech by the placement of the nose of the dog 1 cm above the salt solution tub, and when the leech was hanging out of the nostrils, grasped it with the help of forceps after several attempts. Endoscope-guided removal of nasal leech provides a good success rate and avoids any unnecessary complications in the study. Alive and intact leeches were successfully retrieved with a smaller number of attempts. Hou et al. (5) also removed living leech from the nasal cavity by the rhinoscopy-guided method in humans. They also found the hyperemic nasal mucosa along with the presence of blood. Similar findings were also observed in the present study. The movement of leech over the sensitive nasal mucosa resulted in sneezing and the attachment sites of suckers resulted in multiple bleeding spots.

The wait-and-watch policy after irrigation of saline solution into the nasal cavity is a more time-consuming method (6). The tropical areas like Mediterranean countries, such as Africa and Asia, had a higher incidence of nasal leech infestation (6). From the above case study, it was concluded that video-otoscope guided retrieval of nasal leeches was convenient method without any major complication. It facilitated direct visualization of nasal leech and its retrieval from deeper parts of the nasal cavity in an easier manner.

## Learning Points/Take-Home Messages

- Nasal leech infestation can lead to intermittent epistaxis, sneezing and reverse sneezing.
- The rhinoscopy technique provides the only means of direct visual diagnosis for nasal leech infestation.
- Rhinoscopy allows precise retrieval of nasal leeches.
- Endoscope-assisted removal of nasal leech reduces the complications and duration of the retrieval procedure as compared to the traditional method of leech removal.

## Patient's Perspective

The patient (dog) has been relieved from the nasal leech which irritated and sucked out the blood of the dog. The dog appeared to be healthier and there was no further problem of epistaxis or sneezing. The owner of the dog was satisfied with the treatment.

## Data Availability Statement

The original contributions presented in the study are included in the article/supplementary material, further inquiries can be directed to the corresponding author/s.

## Ethics Statement

Ethical review and approval was not required for the animal study because the present communication is a case study about the findings of nasal Leech in a dog, its confirmation by endoscopy and subsequent retrieval by the help of grasping forceps under endoscopic guidance. The case was done after the primary complaint from the owner showing some upper respiratory disease condition, so no ethical permission was sorted as the findings are the result of the routine clinical case under treatment under a well-established clinical setting in a state veterinary college. Written informed consent was obtained from the owners for the participation of their animals in this study.

## Author Contributions

MS and AmK performed the endoscopic procedure. ST actively assisted the endoscopic procedure and helps in manuscript writing. AdK was the anesthesiologist during the procedure. RW facilitated the documentation and recording of the case along with manuscript writing. All authors contributed to the article and approved the submitted version.

## Conflict of Interest

The authors declare that the research was conducted in the absence of any commercial or financial relationships that could be construed as a potential conflict of interest.

## Publisher's Note

All claims expressed in this article are solely those of the authors and do not necessarily represent those of their affiliated organizations, or those of the publisher, the editors and the reviewers. Any product that may be evaluated in this article, or claim that may be made by its manufacturer, is not guaranteed or endorsed by the publisher.
